# Prognostic significance of miR-194 in endometrial cancer

**DOI:** 10.1186/2050-7771-1-12

**Published:** 2013-02-18

**Authors:** Haiyan Zhai, Mihriban Karaayvaz, Peixin Dong, Noriaki Sakuragi, Jingfang Ju

**Affiliations:** 1 Translational Research Laboratory Department of Pathology Stony Brook Cancer Center, Stony Brook University School of Medicine, BST-2, L-9, Room 185, Stony Brook, NY, 11794, USA; 2 Department of Gynecology, Hokkaido University Graduate School of Medicine and School of Medicine, Hokkaido University, Sapporo, Japan

**Keywords:** MiR-194, Endometrial cancer

## Abstract

Endometrial cancer (EC) is the leading malignant tumor occurring in the female genital tract and some subtypes are highly invasive and metastatic. miRNAs are small non-coding RNAs that have a broad impact on cancer progression. In particular, miR-194 regulates epithelial to mesenchymal transition (EMT) by suppressing the expression of BMI-1 in EC. In this retrospective study, the clinical significance of miR-194 was investigated in archival EC specimens. We extracted total RNA from thirty-two EC samples and quantified the expression level of miR-194. We discovered that the expression level of miR-194 was significantly (P = 0.03) lower in type I EC patients with more advanced stage. In addition, patients with higher miR-194 levels have better prognosis than those with lower miR-194 levels (P = 0.0067; Cut-off value of miR-194 = 0.3). These results indicate that miR-194 has potential to serve as prognostic biomarker for EC patients.

## Background

Endometrial cancer (EC) is the most frequent malignant tumor occurring in the female genital tract in the United States [1]. Generally EC cases can be classified into two broad categories based on their clinical and pathological features. Around 80% of all the EC cases are Type I EC, endometrioid EC (EEC), which are estrogen-dependent. Most of EEC cases are low stage and low grade, and have a better prognosis [2]. In contrast, Type II EC cases are not dependent on estrogen and have more cases in advanced stages, especially serous carcinomas (ESC) or clear-cell carcinomas (CCC), which constitute approximately 10% of all the EC cases. These types of EC are shown to be more aggressive, and have a poor prognosis [2]. Although overall 5-year survival rate of EC patients is relatively higher than those of other gynecologic cancers, around 80% among all stages, certain histological types of endometrial cancer are highly invasive and easily metastatic with low survival rate [3]. Thus there is an emerging need for highly sensitive and specific molecular prognostic biomarkers besides the pathological diagnosis based on the morphological alterations, to better predict the outcome of EC.

In the past 10 years, small regulatory RNAs have gained enormous interests in cancer research. microRNAs (miRNAs) are a class of non-coding RNA molecules, 18–25 nucleotides in length, that regulate the expression of their target genes by translational arrest or mRNA cleavage mostly via direct interaction with the 3^′^-UTRs of the target mRNAs [4,5]. Base pairing between at least six consecutive nucleotides within the 5^′^-seed of the miRNA with the target site on the mRNA is reported to be a minimum requirement for the miRNA-mRNA interaction [6]. miRNAs have been found to regulate many cellular processes including apoptosis [7-10], differentiation [5,11,12] and cell proliferation [7,12-14]. Several reports indicate that aberrant expression levels of certain miRNAs in both plasma and cancer tissue correlates with the EC patients’ survival rate, which can be used as predictive biomarkers [15-17].

Recently the expression pattern and function of miR-194 has been widely studied in various cancers but remains controversial. miR-194 was found to be up-regulated in cancerous tissue when compared to adjacent normal tissue in the esophagus, and its expression level is higher in adenocarcinoma tissue than in squamous cell carcinoma [18]. In addition, the overexpression of miR-194 was found in highly metastatic pancreatic ductal adenocarcinoma (PDAC) cell lines [19]. However, other reports discovered that miR-194 level was down-regulated in colon cancer, colorectal liver metastases, liver cancer and nephroblastomas [20-23], indicating the function of miR-194 is dependent on its cellular context. Ectopic overexpression of miR-194 has been shown to enhance the colon cancer angiogenesis *in vivo* through inhibition of its target – thrombospondin-1 [24]. In breast cancer cells, the inhibition of HER2 by monoclonal antibody led to upregulation of miR-194. Overexpression of miR-194 induced the inhibition of its target, talin 2, and in turn reduced cell migration and invasion [25]. Similarly, studies from our group have demonstrated the functional significance of miR-194 in endometrial cancer by suppressing BMI-1 expression to regulate epithelial to mesenchymal transition [26]. Our results show that ectopic expression of miR-194 in EC cells inhibited its target, BMI-1, to prevent EMT and inhibit tumor invasion. Additional miR-194 targets involved in EMT or metastasis were reported in liver cancer, including N-cadherin, RAC1, heparin-binding epidermal growth factor-like growth factor (HBEGF), type 1 insulin-like growth factor receptor (IGF1R) [20].

In this study, we investigated the clinical significance of miR-194 in EC. We quantified the expression level of miR-194 in archival formalin fixed paraffin embedded (FFPE) EC clinical specimens. The expression levels of miR-194 were then correlated with clinical parameters such as disease stage, disease type, and patient survival. Our results show that miR-194 is significantly associated with histology Type I EC. Kaplan-Meier survival analysis revealed that high levels of miR-194 are associated with a longer survival. As a result, miR-194 may have a potential as a novel prognostic biomarker for EC patients.

## Results and discussion

### Expression level of miR-194 is inversely correlated with cancer stage in type I EC

**Figure 1:**
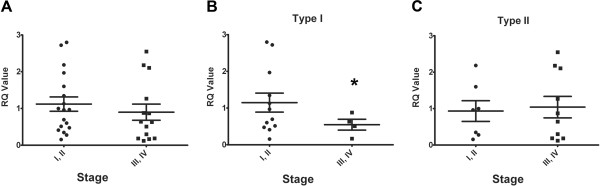
**miR-194 expression level decreased in type I EC samples of higher stage.** The miR-194 expression level in EC samples was categorized based on pathology stage. There is no significant difference between early stage (I, II) and late stage (III, IV) in overall EC samples (**A**, P = 0.2295) or type II EC (**C**, P = 0.7956). However miR-194 expression level was lower in late stage than early stage in type I EC (**B**, P = 0.0323).

In this study, we needle dissected EC tissues from thirty-two FFPE EC specimens and extracted RNAs to quantify the relative expression level of miR-194. We then seperated the patients into two clinical groups based on their pathology reports, early stage (stage I and II) and late stage (stage III and IV). Our analysis results show a trend that the miR-194 level was lower in late stage EC samples (Figure 1A) but not statistically significant (P = 0.2295). To determine whether this expression pattern was associated with histology type, we further seperated the patients based on their histology type (type I and II EC) and clinical stage. We found that miR-194 expression level was significantly downregulated in late stage type I EC samples (Figure 1B, P = 0.0323), however no significant correlation was found in type II EC samples (Figure 1C).

It has been reported that miR-194 regulates cell migration and invasion in various cancer types, including EC, breast cancer and liver cancer [20,25,26]. Especially in EC, the target of miR-194 was found to be BMI-1 [26], an important oncogene regulating EMT [27]. We have further demonstrated in our previous studies the functional significance of miR-194 in regulating EMT transition in endometrial cancer by regulating the expression of E-cadherin and vimentin [26]. Overexpression of BMI-1 has been found in many human cancers, including lung cancer, prostate cancer, breast cancer, ovarian cancer and EC [28-32]. The Knockdown of BMI-1 in EC cell lines showed similar phenotypes as ectopic expression of miR-194, which led to upregulation of E-cadherin, downregulation of Vimentin and impairment in cell invasion [26]. Consistent with these results, our data showed lower expression of miR-194 in stage III and IV type I EC samples when compared to stage I and II samples, indicating miR-194 was inversely correlated to the cancer aggresiveness. However we did not find any difference in miR-194 expression level in type II EC samples or overall EC samples. One possible reason is that our clinical samples consisted of both type I and II EC samples, which have distinct clinical features. And the seventeen type II EC samples consisted of four different histology subtypes, which contributed to the sample complexation. Larger EC patient cohorts are clearly needed in future studies to fully validate our findings. In addition, due to the limitation of FFPE tissue, we were not able to quantify the protein expression levels of key known miR-194 targets such as BMI-1 and TMP1.

### Expression level of miR-194 correlated with EC patients’ suvival time

**Figure 2:**
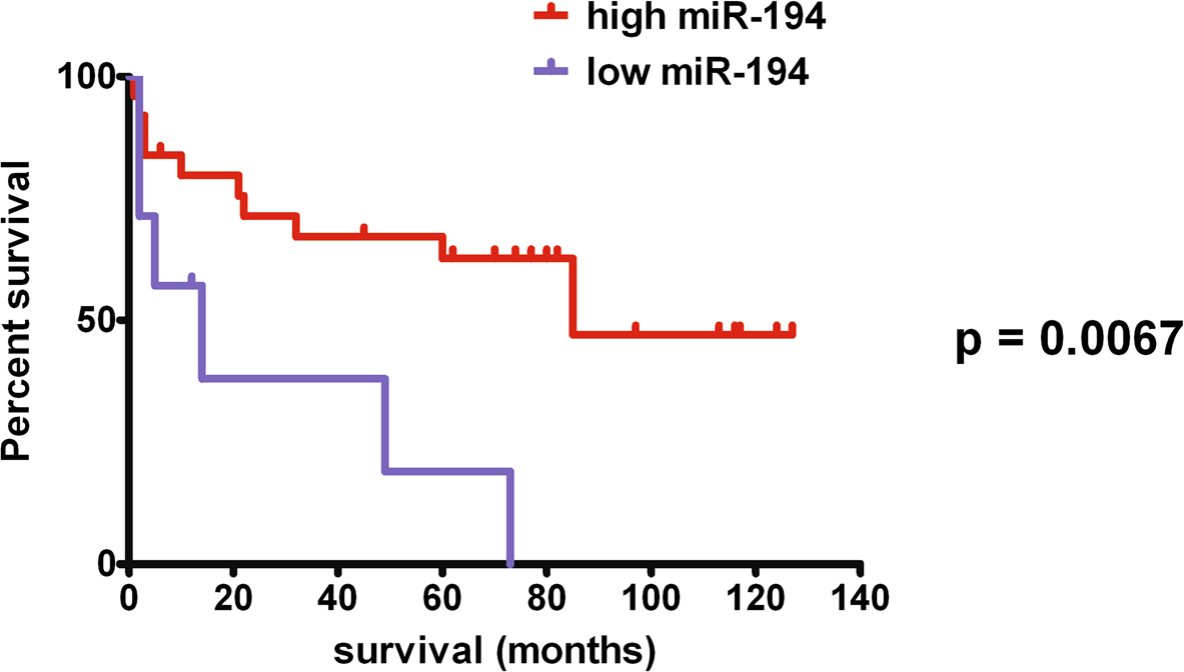
**miR-194 expression level can be used as a prognostic biomarker for EC patients.** Kaplan-Meier survival analysis of EC patients based on miR-194 expression level. The cut-off expression value of miR-194 = 0.3 (P = 0.0067).

Since we show that miR-194 is inversely correlated with EC aggressiveness, we reason that miR-194 expression level may affect EC patients’ survival. EC patient survival was analyzed by Kaplan-Mirer survival analyses with miR-194 expression cut-off values as 0.3 (Figure 2). We found that the median survival time of EC patients with low miR-194 levels (less than 0.3) was 14 months, which was significantly shorter than those with higher miR-194 levels (greater than 0.3), 85 months. The overall survival rates of these two groups were significantly different (P = 0.0067), which was independent of the histological type. This is highly consistent with the previous reports that elevated miR-194 target BMI-1 expression is associated with increased tumor invasion and metastasis in endometrial cancer [26].

As previous reported, aberrant expression of miRNAs was observed in most tumor types, and multiple clinical screenings showed that miRNAs have a potential to serve as prognostic biomarkers in cancers [33-35]. Moreover, the superior stability of miRNAs in FFPE tissues and various body fluids (plasma, serum, etc.) further facilitates the clinical utility of miRNAs [36,37]. Our previous studies found that miR-205 was overexpressed in EC tissue compared to adjacent normal tissues, and expression levels of miR-205 were significantly inversely correlated with patient survival [16]. In this study, we discovered miR-194, a critical regulator of EMT transition by suppression of BMI-1, was inversely correlated with patient survival rate, implicating miR-194 as a new candidate prognostic biomarker in EC patients.

## Conclusion

Since miRNAs can regulate the expression of multiple targets, they have a broader impact on cancer progression. Due to their superior stability in FFPE tissue, they can be used as diagnostic and prognositic biomarkers for cancer patients. In this study we discovered that the miR-194 expression level was down-regulated in late stage, type I EC patients. The inverse correlation between miR-194 expression level and EC patients’ survival time was independent of histological subtype. These results suggested that miR-194 has potential to serve as a prognostic biomarker for EC patients. Future studies with large multi-center patient cohorts are needed to fully validate the potential of miR-194 as a prognostic biomarker in EC.

## Methods

### Patients and Samples

Thirty-two endometrial cancer patients, who underwent hysterectomy at Stony Brook University Medical Center, Stony Brook, NY, USA, were selected from 1995 to 2010. Patient consent forms were obtained from each patient according to the policies of Institution Review Board. Patient clinical information was provided by the Cancer Registry of Stony Brook University Medical Center, which reported data about patient age, sex, treatment, tumor recurrence and survival for up to 15 years. The characteristics of these patients are shown in Table 1. Representative tissue blocks from each case were assembled from the archival collections of the Department of Pathology, and used for subsequent analysis.

**Table 1:** Clinical features of the 32 endometrial cancer patients

**Characteristics**	**Frequency**	**Percentage (%)**
**Mean age in years (range)**	69 (49–86)	
**Histology**		
**Type I EC** Endometrioid carcinoma	15	46.9
**Type II EC**	17	53.1
Serous carcinoma	8	25
Clear cell carcinoma	5	15.6
Malignant mixed mullerian tumor	3	9.4
Undifferentiated carcinoma	1	3.1
**TNM Stage**		
**Type I EC**		
I	11	34.4
II	0	0
III	1	3.1
IV	3	9.4
**Type II EC**		
I	6	18.8
II	1	3.1
III	4	12.5
IV	6	18.8
**Survival (Months)**		
Mean (range)	52 (1–127)	
0-40	14	43.8
40-80	9	28.1
>80	9	28.1

### RNA Isolation

From the archival FFPE tissues, areas of endometrial cancer were identified using the corresponding hematoxylin and eosin (H&E) stained sections and cores measuring 1.5 mm in diameter and 2 mm in length (approximately 0.005 g) were extracted. Then the samples were deparaffinized with xylenes, hydrated by using decreasing concentrations of ethanol, and digested with proteinase K. Total RNA was isolated with Trizol reagent according to the manufacture’s protocol (Life Technologies, CA, USA).

### Real time qRT-PCR analysis of miRNA expression

All reagents for real-time qRT-PCR were ordered from Life technologies. For quantification of miR-194, 10 ng of RNA was used as a template and cDNA was synthesized with high capacity cDNA synthesis kit and miRNA-specific primers (miR-194 and internal control RNU44). Then the cDNA templates were mixed with gene-specific primers for miR-194 and RNU44, and Taqman 2x universal PCR master mix. Applied Biosystems 7500 Real-Time PCR machine was used for qRT-PCR and programmed as: 95°C, 10 minutes; 95°C, 15 seconds; 60°C, 1 minute, which were repeated for 40 cycles. Fluorescent signals from each sample were collected at the endpoint of every cycle, and the expression level of genes and miR-194 was calculated by Δ*C*_T_ values based on the internal control, normalized to one control sample from normal endometrium and plotted as relative value (RQ).

### Statistical Analysis

All statistical analyses were performed using GraphPad Prism software 5.0. Kaplan-Meier survival curves were generated to examine the relationship between the expression levels of miR-194 and patients’ survival rate. The statistical significance between two groups was determined using unpaired Student’s *t*-test with Welch’s correction. Data were expressed as mean ± standard error of the mean (SEM). The statistical significance is described in figure legends.

## Competing interests

The authors declare no conflict of interest.

## Authors’ contributions

HZ and JJ, designed the project; HZ, MK, performed the experiments; HZ, PD, NS, analyzed the data; HZ and JJ, wrote the manuscript. All authors read and approved the final manuscript.
